# Circular at the very beginning: on the initial genomes in the RNA world

**DOI:** 10.1080/15476286.2024.2380130

**Published:** 2024-07-17

**Authors:** Yufan Luo, Minglun Liang, Chunwu Yu, Wentao Ma

**Affiliations:** a Hubei Key Laboratory of Cell Homeostasis, College of Life Sciences, Wuhan University, Wuhan, China; b College of Computer Sciences, Wuhan University, Wuhan, China

**Keywords:** The origin of life, the RNA world, circular chromosome, early evolution, computer simulation

## Abstract

It is likely that an RNA world existed in early life, when RNA played both the roles of the genome and functional molecules, thereby undergoing Darwinian evolution. However, even with only one type of polymer, it seems quite necessary to introduce a labour division concerning these two roles because folding is required for functional molecules (ribozymes) but unfavourable for the genome (as a template in replication). Notably, while ribozymes tend to have adopted a linear form for folding without constraints, a circular form, which might have been topologically hindered in folding, seems more suitable for an RNA template. Another advantage of involving a circular genome could have been to resist RNA’s end-degradation. Here, we explore the scenario of a circular RNA genome plus linear ribozyme(s) at the precellular stage of the RNA world through computer modelling. The results suggest that a one-gene scene could have been ‘maintained’, albeit with rather a low efficiency for the circular genome to produce the ribozyme, which required precise chain-break or chain-synthesis. This strict requirement may have been relieved by introducing a ‘noncoding’ sequence into the genome, which had the potential to derive a second gene through mutation. A two-gene scene may have ‘run well’ with the two corresponding ribozymes promoting the replication of the circular genome from different respects. Circular genomes with more genes might have arisen later in RNA-based protocells. Therefore, circular genomes, which are common in the modern living world, may have had their ‘root’ at the very beginning of life.

## 1. Introduction

It is now widely accepted that in the early evolution of life, there was a stage called the ‘RNA world’ [1–4], during which RNA served as the carrier of both genes and functions. Indeed, to guarantee the capability of undergoing Darwinian evolution, which is essential for a life form [5–7], the genetic and functional features are both indispensable; on the other hand, it seems quite unlikely that two different types of polymers – one for heredity and the other for functionality (with a ‘information linkage’ in between), like DNA and proteins in modern life – could have emerged simultaneously at the start. Due to its logical reasonability as well as accumulating evidence supporting the idea [3,4,8], the RNA world scenario appears quite convincing and is now guiding many studies in the field of the origin of life, both from experimental and theoretical aspects [9–12].

Moreover, in the context of recent advances in prebiotic synthesis [13], particularly those concerning nucleotides [14,15], it seems likely that the RNA world simply represented the earliest stage of life (disregarding the ‘metabolism first’ hypothesis or similar theories postulating the initial emergence of life in the absence of Darwinian evolution, see Ref [8] for an in-depth comment). Then the question arises: how could the RNA world have started, for example, in ‘a pool of nucleotides’? [9].

Modern life is based on cellular forms (except virus and viroids, which live parasitically, relying on other cellular organisms). However, considering the principle of simplicity in the origin problem (‘the simpler, the more likely to have emerged de novo’), it has been hypothesized that there was a ‘naked’ phase at the beginning of the RNA world [10,16,17]. That is, it is initially RNA molecules themselves that acted as the units of Darwinian evolution (i.e. Darwinian entities), and RNA-based ‘protocells’ emerged later – marking the ‘first major transition’ in the evolutionary history [18–20]. Here, we focused our modelling on the precellular naked phase – therein, how could RNA have played the dual roles to ensure Darwinian evolution?

For an RNA molecule to act as a ribozyme (functional molecule), effective folding to reach an appropriate structure is important. However, to act as a good template in replication (‘genome’ for heredity), folding is unnecessary and folding into a compact form is even rather unfavourable. Then, we asked: though with only one type of polymer, could some form of labour division be taken among the RNA molecules? In fact, in modern life, enzymes and ribozymes typically adopt a linear form as a polymer, whereas circular DNA/RNA genomes are ubiquitous (e.g. bacteria, archaea, mitochondria, chloroplasts, viroids and some virus). Indeed, there is evidence that circularization of a linear RNA will interfere with its functional folding [21] – for example, the hammerhead ribozyme within the circular genome of a viroid can only function in a linear form when participating the rolling circle replication of the genome [22] (i.e. when residing in the circular genome, the ribozyme cannot fold into a ‘correct’ structure). Beside the advantage of potentially acting as a better template, a circular form can protect the genome from end-degradation, especially considering the chemical instability of RNA molecules (see Discussion for a detailed explanation).

As another clue indicating that we should consider the circular form of RNA at this early phase, circularization of linear RNAs seems actually inevitable. It has long been appreciated that a polymer might undergo intramolecular circularization when grown to sufficient length, so that its two ends can come within close proximity [23]. For RNA, such intramolecular end-to-end ligation turns out to be rather ready, as a ‘parallel’ reaction of the intermolecular end-to-end ligation [24,25]. In other words, circular RNA should be common in the early phase and may have inevitably taken part in the ‘primordial’ Darwinian evolution occurring then.

**Figure 1.**
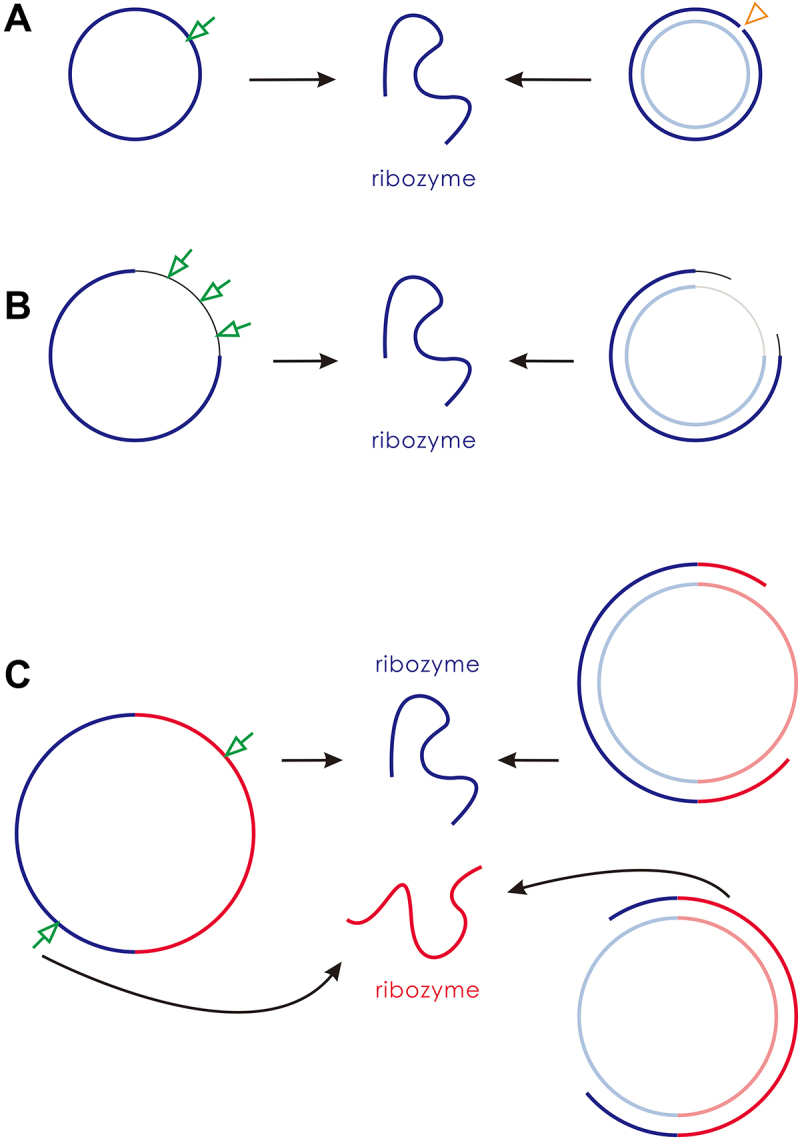
Circular genomes and their ways to generate linear ribozymes in the beginning of the RNA world. (A) A ‘compact’ one-gene genome. Blue lines represent the ribozyme sequence and light-blue lines represent the corresponding complementary sequence. The ribozyme must be generated through accurate breaking (the arrow) of the sense chain or through ‘accurate RNA synthesis’ on the anti-sense chain template – i.e. starting at the correct position and timely dissociation of the produced linear sense chain before its circularization (the triangle). (B) A one-gene genome including a ‘noncoding’ sequence. Thin black lines represent the noncoding sequence, and thin grey lines represent its complement. The ribozyme could be generated by any chain-breaking events within the non-coding sequence (the arrows), or by any instances of dissociation of the produced linear sense chain (providing it already includes the ribozyme sequence) before its complete circularization. (C) A two-gene genome. Red lines represent the sequence of the second ribozyme, and light-red lines represent its complement. One ribozyme may be generated by breaking the circular sense chain at the side of the other ribozyme’s region, or the dissociation of the produced linear sense chain comprising a complete sequence of the ribozyme.

According to the implication of simplicity on the origin problem, we may assume that the RNA world started with one functional ribozyme. However, when we envisioned a corresponding one-gene circular genome, the thing became somewhat ‘delicate’. How can the linear ribozyme be ‘produced’ from the circular genome? Figure 1A shows the two possible ways: one is the ‘accurate breaking’ of the ‘sense’ chain; the other is the ‘accurate synthesis’ of the ribozyme on the ‘antisense’ chain (including the starting of synthesis at correct position and the timely dropping from the template before the ultimate completion of circularization). Both of them appear quite difficult in such an early scene of evolution. In fact, for this early scene, in a context of ‘random breaking’ and ‘random synthesis’, most of the products derived from the circular genome would not be the ribozyme (thus ‘useless’). Then, could such an inefficient mode to generate the ribozyme be effective enough to support the spread of the one-gene genome? With this question in mind, we set out on our modelling work.

## 2. Results

### 2.1. About the model

We conducted the computer simulation using a Monte Carlo model similar to those used in our previous work concerning the naked phase of the RNA world [26–29], but with consideration of circular RNA. The system is a two-dimensional *N* × *N* square grid (with toroidal topology to avoid edge effects). The assumption of a two-dimensional system is in part for simplicity and in part with consideration of the prebiotic milieus like mineral surfaces (with dispersal limitation) for the naked phase of the RNA world, as suggested by quite a lot of studies [30–33]. Molecules are distributed within the grid rooms, including nucleotide precursors, nucleotides, and RNA. In each time step (Monte Carlo step), relevant events may occur to the molecules with defined probabilities. Nucleotide precursors may transform into nucleotides (randomly as A, G, C, or U). Nucleotides may assemble into linear RNAs via random ligation. A linear RNA may turn into a circular one via end-to-end ligation. Both linear RNAs and circular RNAs may conduct template-directed replication by using nucleotides and oligomers as substrates, except that linear RNAs have a lower templating efficiency. A circular RNA may turn into a linear one via the breaking of a certain phosphodiester bond. A linear RNA may also break into smaller fragments. A nucleotide may decay into a nucleotide precursor. A nucleotide residue at the end of a linear RNA may also decay into a nucleotide precursor. A linear RNA molecule containing a characteristic sequence is assumed to have a special function (i.e. may act as a ribozyme). Molecules may also move into an adjacent grid room. Refer to Table 1 for descriptions of the associated parameters and see Methods for detailed explanations.

**Table 1.** Parameters used in the computer simulation.

	Descriptions	Default Values
**Probabilities**		
*P*_*AT*_	An RNA template attracting a substrate (nucleotide or oligomer)	0.5
*P*_*BB*_	A phosphodiester bond breaking within an RNA chain	5 × 10^−6^
*P*_*EL*_	The end-to-end ligation of an RNA chain (circularization)	1 × 10^−7^
*P*_*FP*_	The false base-pairing when an RNA template attracts a substrate	0.001
*P*_*MN*_	The movement of nucleotides	0.002
*P*_*MNP*_	The movement of nucleotide precursors	0.01
*P*_*ND*_	A nucleotide decaying into its precursor	0.05
*P*_*NDE*_	A nucleotide residue decaying at RNA’s chain end	0.001
*P*_*NF*_	A nucleotide forming from its precursor (non-enzymatic)	0.005
*P*_*NFR*_	A nucleotide forming from its precursor catalysed by NR	0.9
*P*_*RL*_	The random ligation of nucleotides and RNAs	1 × 10^−7^
*P*_*SP*_	The separation of a base pair	0.5
*P*_TL_	The template-directed ligation (non-enzymatic)	0.01
*P*_*TLR*_	The template-directed ligation catalysed by REP	0.9
**Others**		
*N*	The system is defined as an *N* × *N* grid	30
*T*_*NPB*_	Total nucleotide precursors introduced in the beginning	1 × 10^5^
*T*_*REP*_	The times for a REP to function in a time step	10
*T*_*NR*_	The times for an NR to function in a time step	10
*F*_*DA*_	The factor concerning the *de novo* attraction of a substrate	5
*F*_*LT*_	The factor for a linear RNA acting as a template	0.5
*CS*_REP_	The characteristic sequence of REP	GAGUCUCU
*CS*_*NR*_	The characteristic sequence of NR	UGAUGCAG
*CS*_*CT*_	The characteristic sequence of the control RNA	ACGAACUG

Notes: The probabilities are listed in alphabetical order. The simulation cases shown in the present paper adopt the default parameter values, unless being explicitly stated otherwise. Note that the outcome of a simulation is generally robust against moderate alteration of the parameter values. See Methods for a detailed explanation of the parameters and guidelines for their configuration.

In the beginning of a simulation, a certain number of nucleotide precursors are introduced into the system. As time goes on, nucleotides and RNA would emerge. On the other hand, the degradation of RNA and nucleotides may end in nucleotide precursors. In summary, the total materials of the system are constant. RNA sequences within the system are competing for the limited materials. Potentially, an RNA ‘species’ – with a specific sequence may spread (become thriving) in the system by virtue of its function (favouring its replication). Notably, with a ‘information resolution’ at the nucleotide level, the model is intrinsically suitable for the investigation of the early Darwinian evolution, which relied essentially on the sequence-function connection of RNA.

### 2.2. The spread of a one-gene circular genome

An RNA species catalysing the template-directed synthesis (thus the RNA replication) has long been suggested to have been the first ribozyme emerging in the RNA world, usually referred to as an ‘RNA replicase’ [9,34–37] (here ‘REP’ for short). Although experimental studies have not achieved such a ribozyme in a complete sense, we can see a constant progress towards this direction over the past three decades [38–44]. Computer simulation has demonstrated that RNA-like ‘replicators’ could spread at the naked phase by virtue of replicase-like function (via favouring its own replication due to limited dispersal) [45]. A previous simulation study of ours suggested that the RNA replicase emerging first may have been a simple template-directed ligase, i.e. a ribozyme catalysing the ligation of substrates (nucleotides and oligomers) aligned adjacently on the template [28]. Therefore, here we considered such a ribozyme and its corresponding gene in our investigation of the one-gene circular genome.

**Figure 2.**
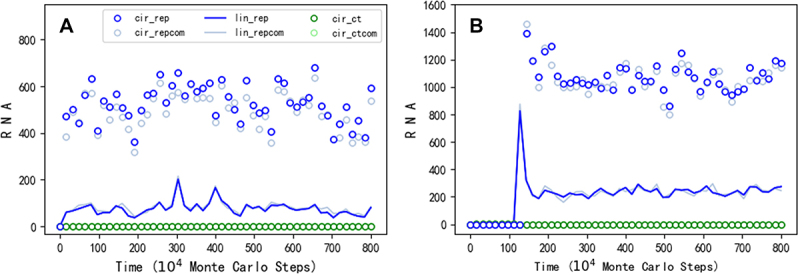
The spread of a circular genome containing only the REP gene. Legends: cir_rep – circular RNA containing the REP sequence; cir_repcom – circular RNA containing the complementary sequence of the REP; lin_rep – linear RNA containing the REP sequence; lin_repcom – linear RNA containing the complementary sequence of the REP; cir_ct – circular RNA containing the control sequence; cir_ctcom – circular RNA containing the complementary sequence of the control. (A) At step 1 × 10^4^, 50 linear RNA molecules with the REP sequence and the same number of linear RNA molecules with the control sequence are inoculated into the system (at locations which are randomly chosen, the same below). (B) One linear RNA molecule with the REP sequence and one linear RNA molecule with the control sequence are inoculated into the system every 1 × 10^4^ steps, until step 1.23 × 10^6^, when the circular REP genome begins to spread.

First, we wanted to know whether a circular RNA comprising the sequence of REP could spread in our model system, under the restriction of inefficient ribozyme-production as mentioned above (Figure 1A). After introducing nucleotide precursors in quantity of *T*_*NPB*_ (see Table 1 for descriptions of parameters) in the beginning, at step 1 × 10^4^, 50 linear RNA molecules with the sequence of REP and 50 linear control RNA molecules (without function) are inoculated into system. Then, circular RNA with the REP sequence appears (via intramolecular end-to-end ligation, with the probability *P*_*EL*_) and subsequently spreads in the system, whereas the control species cannot spread (see Figure 2A for a typical case). Notably, the circular RNA with the REP sequence (blue circles) is roughly equal in number to its complement (light blue circles), and the linear RNA with the REP sequence (the blue line) is also roughly equal in number to its complement (the light blue line). Such a feature, actually present in all simulation cases here, is in accord with the underlying mechanism of template-directed replication based on base-pairing, which is appropriately reflected in our model with a ‘information resolution’ at the nucleotide level.

Here, to avoid the influence of accidental RNA-degradation events, we inoculated a number of REP molecules initially to investigate the evolutionary dynamics (i.e. the evolutionary process over time). In fact, the outcome, i.e. the spread of the circular RNA containing the REP sequence, already suggests that such a kind of RNA could have emerged *de novo* from the proposed ‘nucleotide pool’. In the reality, the simultaneous appearance of so many REP molecules was obviously impossible. However, one molecule of the REP may have had chances to appear repeatedly, especially considering the great time-scale regarding the origin of life. For example, Figure 2B shows a modelling case that one linear REP molecule and one linear control molecule are inoculated into the system every 1 × 10^4^ steps, and the circular REP genome eventually spreads in the system (after the spread, the periodic inoculation stops).

### 2.3. The roles of the circular genome and the ribozyme

**Figure 3.**
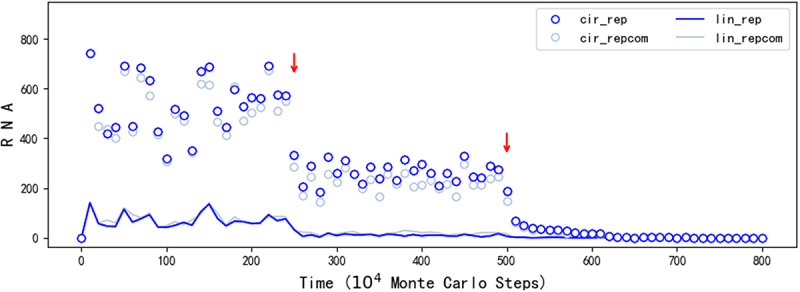
The analysis on the roles of the circular genome and the ribozyme (in regard to REP). The legends are interpreted in the same way as those in Figure 2. The case is just the one shown in Figure 2A, but excludes symbols related to the control sequence, which consistently hover near zero throughout the simulation. This omission prevents their interference with showcasing the linear REP and its complement, both of which exhibit very low levels after the adjustment of parameters (red arrows). At step 2.5 × 10^6^, *F*_*LT*_, which is related to the ability of a linear RNA acting as a template, is altered from 0.5 (default value, see Table 1) to 0 for the linear RNAs containing the REP sequence or its complementary sequence. Then, at step 5 × 10^6^, *P*_*TLR*_, which reflects the catalytic ability of REP, is modified from 0.9 (default value) to 0. See text for a detailed explanation of the analysis.

Firstly, we asked: Could the circular RNA really play the role of genome in the spread of REP? So we turned off the templating capability of the linear RNA with the REP sequence or its complement (*F*_*LT*_ = 0) midway (Figure 3, at step 2.5 × 10^6^). The outcome is the decline of REP – both the circular form and the linear form. In other words, after the turning off, the linear RNA with the REP sequence, which may act as a replicase ribozyme, can only be produced from the breaking of the circular RNA with the REP sequence, or via the copying on the template of the circular RNA with the complement of the REP sequence. Obviously, the spread can sustain, albeit at a lower level. Indeed, the low level of the spread (especially, the number of linear REP is rather low) is understandable considering the inefficient way of a circular genome to produce the corresponding ribozyme (Figure 1A). Simply put, though initially it may have been both the circular form and the linear form that played the role of genome, the circular form can actually play the role of genome alone.

Then, we wanted to confirm: When the circular form plays the role of genome alone, is the spread of REP really attributed to the function of the replicase ribozyme? Indeed, the answer is positive – when we turned off the function of the replicase ribozyme (*P*_*TLR*_ = 0 after step 5 × 10^6^, Figure 3), we saw the complete collapse of the spread.

**Figure 4.**
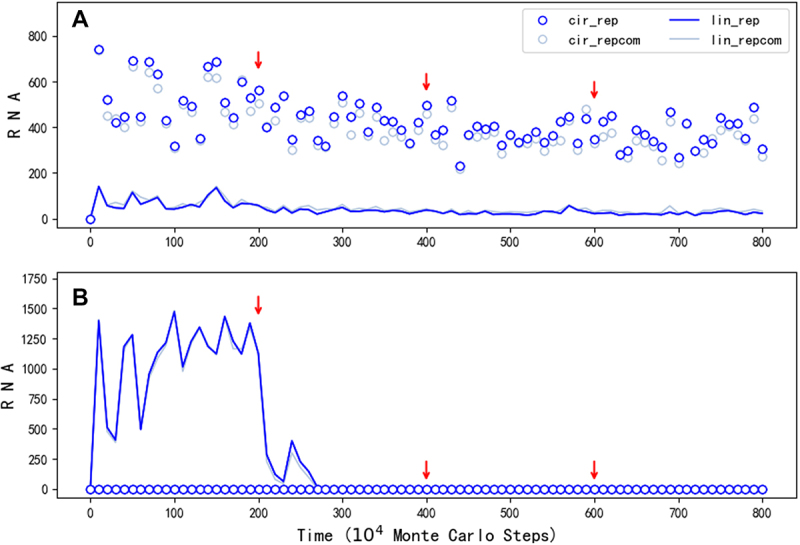
The influence of reducing template capability of the linear REP. The legends are interpreted in the same way as those in Figure 2. The symbols concerning the control sequence, which are nearly at the zero level throughout the simulation, are omitted. (A) The case is just the one shown in Figure 2A, except that *F*_*LT*_ , which represents the template suitability of a linear RNA, is adjusted for the REP ribozyme midway (from the default value 0.5 to 0.2, to 0.1 and finally to 0; see red arrows), the outcome suggests that when the circular RNA can act as a genome, the evolution of the ribozyme towards more complicated folding, (thus with a reduced template capability) would have little impact on its spread as a species. (B) The case is run under the same situations as the case shown in (a), except that *P*_*EL*_, the probability for RNA’s intramolecular end-to-end ligation, is set to 0 and thus no circular RNA can appear in the system. The outcome suggests that when the linear REP ribozyme has to act as a template itself, its evolution towards greater structural complexity may be greatly hindered by the simultaneous loss of its template capability.

Indeed, the modelling suggests that initially both the linear form and the circular form could have played the role of genome (refer to the drop of the spread when turning off the templating capability of the linear form at step 2.5 × 10^6^ in Figure 3), wherein the linear form may have been even more important considering the restriction for the circular form to derive the ribozyme (Figure 1A). But in the long run, it should be the circular form that become more and more important because when the ribozyme evolved towards more efficient – thus with a more compact and complicated structure, the linear form would be more and more unsuitable to act as template. Then, we asked: Can our modelling provide some messages on this tendency? The factor representing the suitability of a linear form RNA to act as template, relative to a circular RNA, is here assumed to be 0.5 as a default value (*F*_*LT*_ = 0.5, refer to Table 1). When the factor is turned down for the linear RNA containing the REP sequence midway, the spread of REP declines only limitedly (Figure 4A). It is worth mentioning that different from the case shown in Figure 3, here this factor is not adjusted for the linear RNA containing the REP’s complement because it seems unreasonable to assume that the complementary sequence of the ribozyme would also adopt a compact and complicated structure. Notably, even *F*_*LT*_ is finally turn off (*F*_*LT*_ = 0 after step 6 × 10^6^), which means that the linear REP completely loses the ability to act as template, the spread is not apparently impacted. As a contrast, if the circular form is assumed to be unable to emerge (*P*_*EL*_ = 0), the turning down of *F*_*LT*_ for the linear form with a REP sequence may be fatal (Figure 4B), because the passing down of the genetic information would be problematic. That is to say, the labour division between the circular form (as the genome) and the linear form (as the ribozyme) should have been more and more clear with the ribozyme’s evolution.

### 2.4. The introduction of a noncoding sequence

Above we have shown that a one-gene circular genome could spread by virtue of the function of the ribozyme it encodes (Figure 3), though the modes for the genome to generate the ribozyme are featured with a strong restriction (Figure 1A). With the restriction, the number of the ribozyme is low and the spread of the genome is limited. Interestingly, we found that this situation may be improved in a simple way. That is, in many cases, a noncoding sequence is introduced into the circular genome, and the restriction is partially released (i.e. the requirement for ‘accurate breaking’ or ‘accurate synthesis’ is no longer so strict, refer to Figure 1B for a schematic).

**Figure 5.**
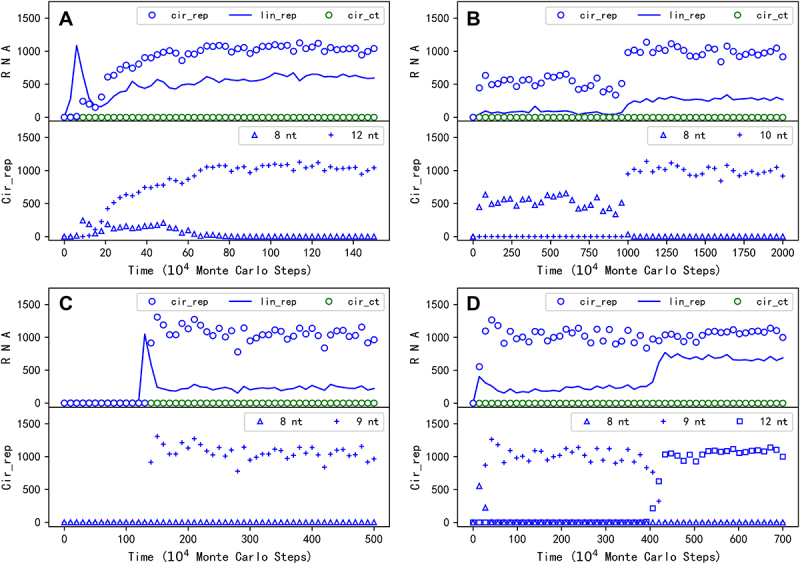
The emergence of circular genomes with a noncoding sequence. The upper panel of a subfigure here is in the same form as a subfigure in Figure 2, except that the complementary sequences of the REP and the control, which are roughly equal in number to their ‘sense chain’ throughout the simulation, are omitted. The lower panel shows the evolution of circular REP genomes with different lengths within the case – corresponding to the legends, for example, ‘8nt’ represents a circular genome only containing the REP sequence (note the default REP sequence is 8nt in length; see Table 1); ‘12nt’ means a circular genome containing a REP sequence plus a 4nt noncoding sequence. (A) The case is in the same situation (i.e. parameter setting) as the case shown in Figure 2A, except with a different random seed. See Figure 6 for some key snapshots concerning spatial distribution of this case. (B) The case is just the one shown in Figure 2A, but with a longer period of simulation time (more Monte Carlo steps). (C) The case is just the one shown in Figure 2B. (D) The case is also in the same situation of the case shown in Figure 2A, except with a different random seed.

**Figure 6.**
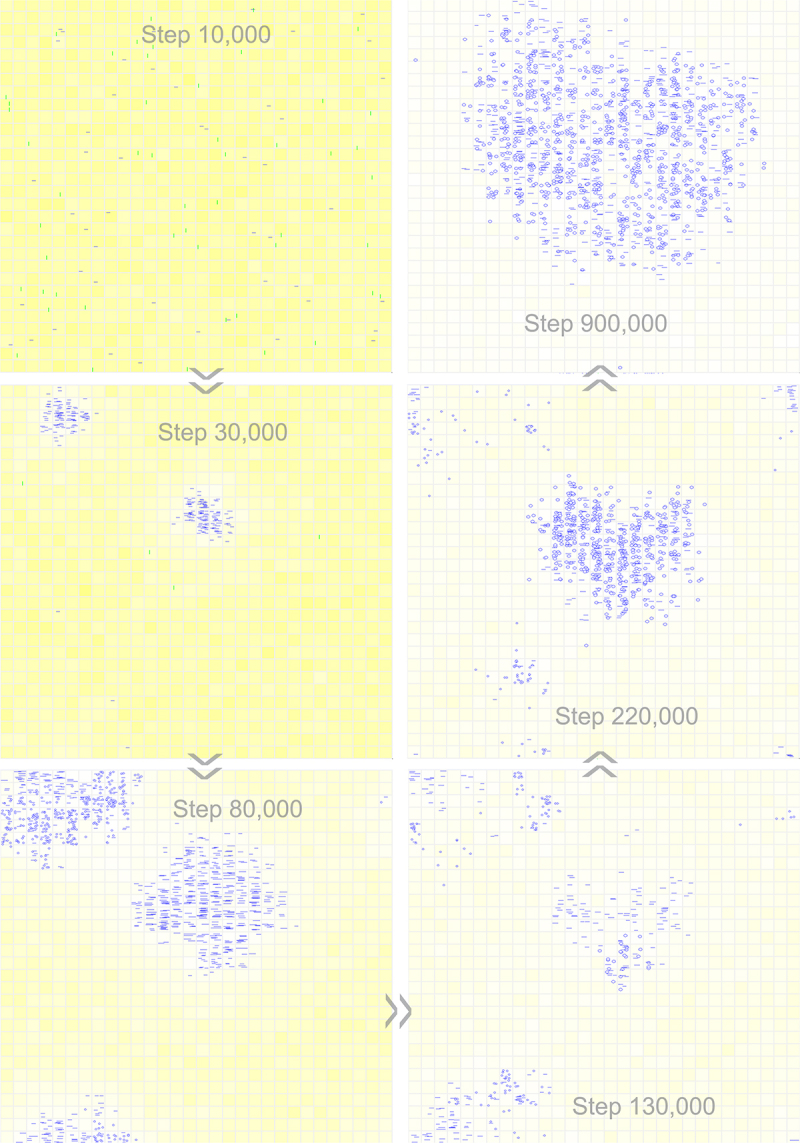
Snapshots showing the emergence of a circular one-gene genome and the subsequent introduction of a noncoding sequence. Raw materials (nucleotide precursors) are shown as yellow background, with colour depth representing their quantity in the corresponding grid room. At step 10,000, 50 linear RNA molecules with the sequence of REP (horizontal blue bars) and 50 linear control RNA molecules (vertical green bars) are inoculated into system (at locations chosen randomly). The snapshot at step 30,000 shows the spread of linear REP molecules (at two different regions). The snapshot at step 80,000 indicates the emergence of the 8nt circular REP genome (small blue circles at the top-left corner, also spreading to the bottom left corner because of the grid’s toroidal topology). The snapshot at step 130,000 indicates the emergence of the 12nt circular REP genome comprising a 4nt noncoding sequence (blue circles at the central part, larger than the 8nt circles). The snapshot at step 220,000 shows the spread of the 12nt genome and the simultaneous decline of the 8nt genome. The snapshot at step 900,000 shows the complete domination of the system by the 12nt genome. See Figure 5A for the evolutionary dynamics of this case.

Figure 5A shows a typical case of such transformation, in which a 12nt genome comprising an 8nt REP sequence plus a 4nt noncoding sequence takes over the compact 8nt REP genome to spread in the system soon after the beginning (see Figure 6 for the spatial distribution snapshots of this case, in which the size of the circular genomes is straightforwardly represented by the size of circles in the pictures). Interestingly, we found that just for the case shown in Figure 2A, when we run the simulation with a larger time scale, a transition from the original 8nt circular genome to a 10nt one occurs (Figure 5B). In this case, it is clear that when a 2nt noncoding sequence is introduced into the circular genome and the restriction is somewhat relieved, the number of the circular genome, as well as the number of the linear ribozymes, reaches a higher level. Additionally, we found that for the case shown in Figure 2B, wherein one REP molecule is inoculated intermittently, the ultimately spreading circular genome is actually 9nt (Figure 5C). That should be the major reason why the level of its spread (Figure 2B) is significantly higher than the case shown in Figure 2A, in which the spreading circular genome is only 8nt in length at that stage. Somewhat strikingly, we even recorded a case in which the circular genome evolves twice – from 8nt to 9nt and then to 12nt (Figure 5D).

No matter how, the introduction of a noncoding sequence, though being apparently disadvantageous for the completion of the genome’s replication, seems to be an acceptable solution to ease the restriction for a one-gene circular genome to generate its corresponding ribozyme (that is, in a context of ‘random breaking’ and ‘random synthesis’, more of the products derived from the circular genome would be the ribozyme). Then, we reasoned that this feature may have favoured the emergence of a second gene into the genome – i.e. via the mutation of the noncoding sequence.

### 2.5. The spread of a two-gene circular genome

In fact, for the ribozymes that may have emerged early in the RNA world, there is another candidate: a ribozyme catalysing the synthesis of nucleotides, i.e. a nucleotide-synthetase ribozyme (‘NR’). This ribozyme can favour its own replication by supplying the building blocks and thus may (in principle of Darwinian evolution) as well have spread in the prebiotic pool – the plausibility of which has been supported by computer modelling [27]. By experimental work, such a synthesis reaction catalysed by RNA has been shown to be feasible [46–48]. Actually, when we assumed that the ribozyme emerging first in the naked phase is an NR, we also saw the spread of the corresponding one-gene circular genome – interestingly, with the introduction of a noncoding sequence as well (Fig. S1). No matter how, here what we are curious about is: if we assume the NR as the second ribozyme emerging in the scene, can the two-gene circular genome spread in the system?

**Figure 7.**
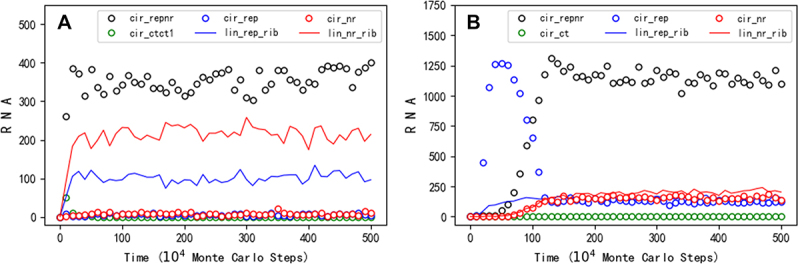
The spread of a two-gene circular genome. Legends: cir_repnr – circular RNA containing both the REP and the NR sequences; cir_rep – circular RNA containing the REP (but not the NR) sequence; cir_nr – circular RNA containing the NR (but not the REP) sequence; lin_rep_rib – linear RNA containing the REP sequence and with a chain length shorter than 1.5 times of the ribozyme’s characteristic sequence (thus able to act as the corresponding ribozyme, see text for details); lin_nr_rib – linear RNA containing the nr sequence and with a chain length shorter than 1.5 times of the ribozyme’s characteristic sequence (thus able to act as the corresponding ribozyme); cir_ctct1 or cir_ct – circular RNA containing a control sequence (see below for details). The complementary sequences of the circular REP-NR and the control, which are roughly equal in number to their ‘sense chain’ throughout the simulation, are omitted. (A) At step 1 × 10^4^, a hundred circular RNA molecules with the sequences of REP and NR (16nt in length, 8nt for one gene), together with the same number of control RNA molecules (also 16nt in length, ‘cir_ctct1’), are inoculated into system. The 16nt control RNA comprises the default 8nt control sequence (refer to Table 1) plus another 8nt sequence (‘AACGCUCG’). (B) At step 1 × 10^4^, a hundred circular RNA molecules with the REP sequence and a noncoding sequence (16nt in length, 8nt for each), together with the same number of control RNA molecules (also 16nt in length), are inoculated into system. The noncoding sequence is ‘UGACGCAG’, which is assumed to be only one residue different from the NR sequence (‘UGAUGCAG’, refer to table 1). The 16nt control RNA comprises an 8nt control sequence ‘ACUGACGU’ plus the noncoding sequence assumed here. The legend ‘cir_ct’ means circular RNA containing the 8nt control. *T*_*NPB*_ = 2 × 10^5^, *P*_*BB*_ = 2 × 10^−5^, *P*_*FP*_ = 0.01, *P*_*NDE*_ = 0.002, and *P*_*NF*_ = 0.001. See Figure 8 for some key snapshots concerning spatial distribution of this case.

Similar to the situation concerning the study of the one-gene genome, firstly, we wanted to know whether a circular RNA comprising both the sequences of REP and NR could spread in the model system. The way of producing the ribozymes from the circular genome is shown in Figure 1C. It may be expected that the two ribozymes, which favour the replication of the circular genome from different aspects (one for the template-directed copying and the other for the synthesis of the building blocks), may aid the two-gene genome to spread in the system. Our simulation turns out to support such a scene. After introducing nucleotide precursors initially, at step 1 × 10^4^, a hundred circular RNA molecules with the sequences of REP and NR (16nt in length, 8nt for each gene), together with the same number of control RNA molecules (also 16nt in length), are inoculated into system. Then, the circular RNA with both the two genes arises and spreads in the system, whereas the control species cannot (see Figure 7A for a typical case). To be clearer, in the figure, the number of the complement chains is not demonstrated, which is actually also roughly equal to the ‘sense’ chains. Additionally, the number of linear REP or NR shown here are the ones shorter than 12nt, as corresponding ribozymes (i.e. lin-rep-rib and lin-nr-rib) – note that a linear RNA comprising the sequence of a ribozyme but with a length of or greater than 1.5 times of this sequence (1.5 × 8nt = 12nt) is in our model assumed to be unable to fold into an appropriate functional structure (see Methods). In fact, the breaking of a two-gene circular genome or the dissociation of a synthesized chain from the genome (refer to Figure 1C) might generate a linear RNA not short enough to be able to fold correctly as a ribozyme, but the short length may be ‘reached’ with the degradation of the RNA (e.g. via end-decay of nucleotide residues).

**Figure 8.**
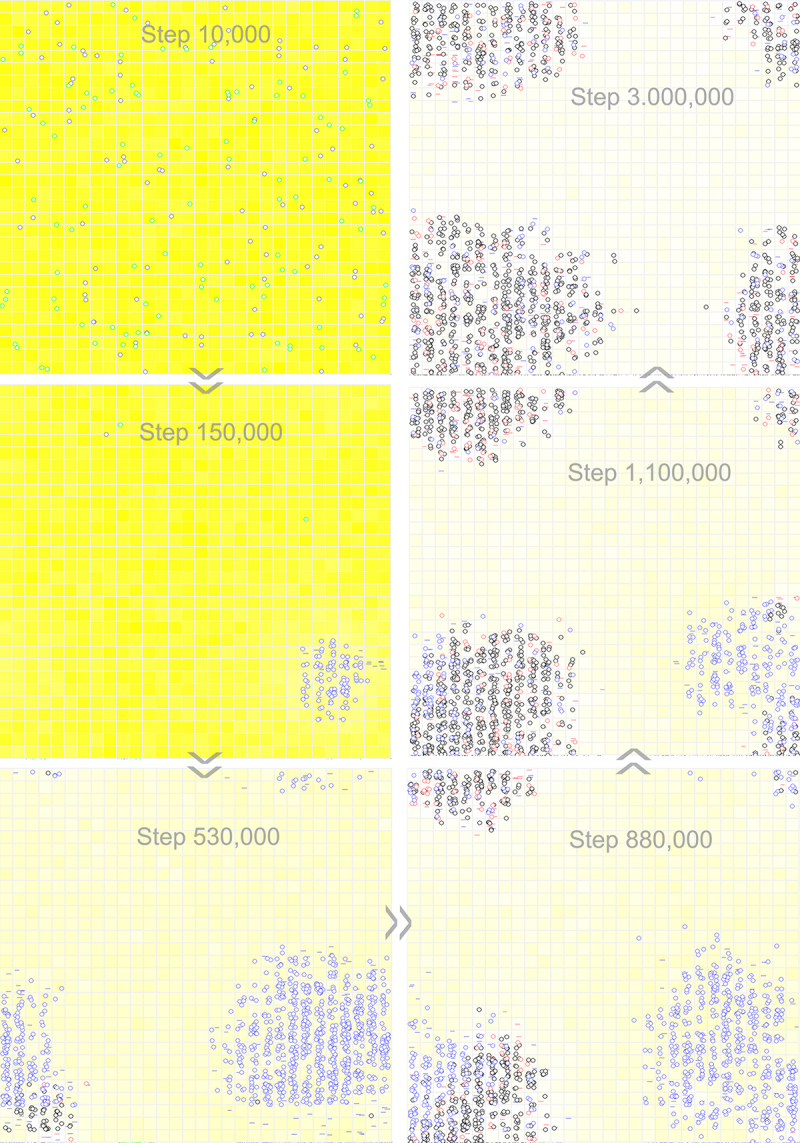
Snapshots showing the deriving of a two-gene circular genome from a one-gene circular genome with a noncoding sequence. Raw materials (nucleotide precursors) are shown as yellow background, with colour depth representing their quantity in the corresponding grid room. At step 10,000, a hundred circular RNA molecules with the REP sequence and a noncoding sequence (blue circles), together with the same number of control RNA molecules (green circles), are inoculated into the system (at locations chosen randomly). The snapshot at step 150,000 shows the spread of the circular genome containing REP. The snapshot at step 530,000 indicates the emergence of the REP-NR circular genome (black circles in the bottom-left region). The snapshot at step 880,000 shows the spread of the REP-NR circular genome. The snapshot at step 1,100,000 shows further spread of the REP-NR circular genome and the simultaneous decline of the genome containing only REP (blue circles). Note that red circles denote the circular genome containing only NR. The snapshot at step 3,000,000 indicates the ‘absolute domination’ of the REP-NR genome in the system. See Figure 7B for the evolutionary dynamics of this case.

Then, we turned to the issue of whether the second gene could emerge through the mutation of the noncoding sequence within a one-gene circular genome. We considered a one-gene circular genome with a REP sequence plus a noncoding sequence. To facilitate the emergence of the second gene, we assumed an NR sequence that differs from the noncoding sequence by only one residue and adopted a relatively higher error rate of template-directed copying (*P*_*FP*_ is adjusted to 0.01 from its default value 0.001, refer to Table 1). After initially introducing nucleotide precursors, at step 1 × 10^4^, a hundred circular RNA molecules comprising the REP sequence and the noncoding sequence (16nt in length, 8nt for each), together with the same number of control RNA molecules (also 16nt in length), are inoculated into system. Then, the one-gene (REP) genome spreads in the system, whereas the control does not. Next, interestingly, we observed the arising of the two-gene (REP-NR) genome alongside the decline of the original one-gene genome (Figure 7B, see Figure 8 for the spatial distribution snapshots). It is noteworthy that in this case, there is a low-level spread of one-gene genome, both for REP and NR (depicted as blue and red circles respectively), which should be largely attributed to mutations in the two-gene genome due to the relatively high error rate assumed for replication.

**Figure 9.**
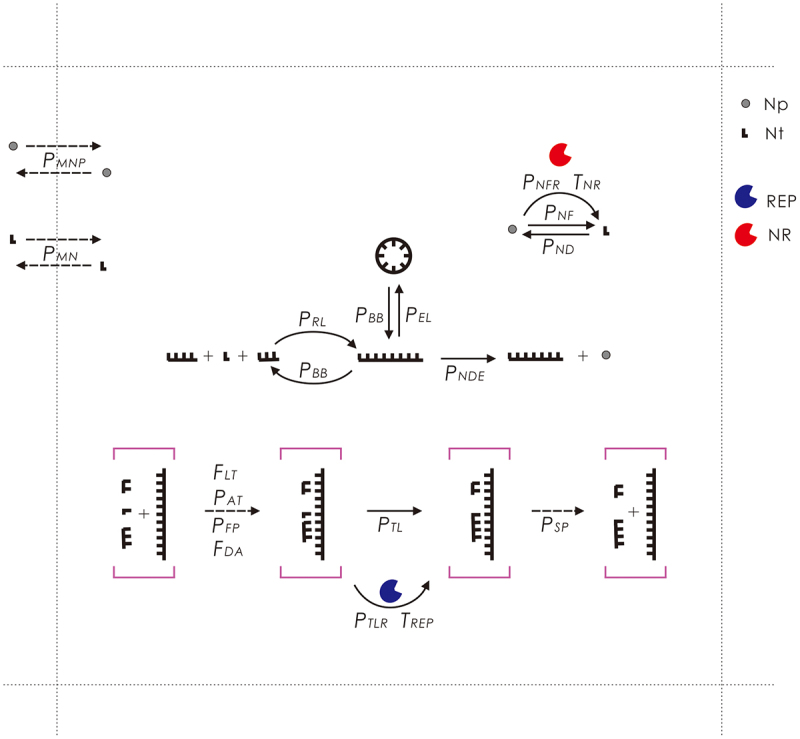
The events occurring in the model and their associated parameters. Legends: Np – nucleotide precursor; Nt – nucleotide; REP – RNA replicase ribozyme; NR – nucleotide-synthetase ribozyme. Solid arrows denote chemical reactions and dashed arrows represent other events. The events concerning the template-directed synthesis are here drawn with respect to a template segment (within purple square brackets), which may belong to either a circular RNA or a linear RNA. Note that the region depicted here represents one grid room in the *N* × *N* grid of the model system. See text for detailed explanations.

Alternatively, we envisioned a scenario akin to the origin of paralogous genes in modern genomes, which involves gene-duplication followed by the emergence of new genes through mutation (because additional copies of the same gene may be ‘of little use’). In the context of our current modelling, for the one-gene REP genome, duplication could occur, for instance, through accidental ligation of two linear REP molecules within the system and subsequent self-circularization. The generation of REP ribozyme from the REP-REP circular genome should be easy, resembling a two-gene circular genome (refer to Figure 1C). Noticeably, the REP-REP genome is even more favoured than the REP-noncoding (REP plus a noncoding sequence) genome because the breaking or synthesis at either side of the duplicated REP regions could lead to a REP ribozyme. Indeed, after inoculating a hundred circular RNA molecules with the REP-REP sequence (at step 1 × 10^4^, alongside control molecules), the genome with the duplicated gene spreads throughout the system. Then, a period of time later, we saw the arising of the REP-NR genome concurrent with the decline of the REP-REP genome (Fig. S2, see Fig. S3 for spatial distribution snapshots). Notably, to facilitate the mutation-driven appearance of NR, here, NR is assumed to differ from REP by only one residue, and as with the previous case (Figure 7B), a relatively higher error rate concerning replication is adopted (*P*_*FP*_ = 0.01).

## 3. Discussion

In the present study, we conducted computer simulation on the initial stage of the RNA world (the precellular phase), examining the plausibility of a circular genome. Our findings suggest that a one-gene circular genome could have spread (Figure 2) even under the stringent constraint of accurately producing the corresponding ribozyme (Figure 1A for a schematic). This restriction could have been alleviated by introducing a noncoding segment into the genome (Figure 5; Figure 1B for a schematic). A second gene might have derived from the noncoding sequence, leading to the appearance of a two-gene circular genome, which could have become thriving by virtue of the two ribozymes it encodes (Figure 7B; Figure 1C for a schematic).

Here, we propose that the requirement of labour division between the genome and functional molecules is the key ‘appeal’ to involve a circular genome, which does not tend to fold structurally. In particular, the division is advantageous in the long run when ribozymes evolved towards higher efficiency – associated with more complicated structures and thus with less templating capability (Figure 4). In fact, such a requirement of labour division has attracted quite a few modelling studies in the area. For example, it was shown that in RNA-based protocells, the ‘trade-off’ between the catalytic activity and the template capability of a ribozyme strand (actually related to the folding degree) may lead to the labour division between the ribozyme strand and its complementary strand, i.e. the former as a functional molecule and the latter as a gene [49]. Such a labour division would result in a quantitative asymmetry of the two strands: the ribozyme strand is more than the gene strand in number. Then, in a later study, it was highlighted that the ribozyme strand may have evolved towards higher catalytic efficiency ‘in order to’ improve the fitness of the protocells containing it, whereas the complementary strand of the ribozyme may have evolved towards higher template capability ‘in order to’ increase the fitness of these molecules (the two strands as one RNA species in the sense of genetic information) inside the protocell [50]. Notably, both the two studies considered the labour division in a context of the cellular phase, whereas here we considered the division that may have already occurred in the naked phase. In addition, both of them thought that the labour division may have been implemented between the two complementary strands of RNA, which is different from our notion here concerning the distinct roles of circular and linear RNA. Note that the trade-off of the ribozyme strand is released with the involvement of the circular genome, and the two complementary strands of the circular genome turn out to be roughly equal in number (as mentioned in Results) – they are actually equivalent in the sense of carrying genetic information. A third example of related modelling work suggested that the labour division may have been implemented with the advent of DNA in the RNA world [51], wherein RNA act as functional molecules and DNA carried the corresponding genes – thereby releasing the trade-off of the ribozyme strand. No matter how, all these studies suggested that the advantage of such a labour division, as a ‘driving force’, may have had important impacts on the evolutionary process in the early stage of life.

Remarkably, a labour division between the genome and ribozymes should have been not only advantageous in the long run but also crucial for resisting parasites (RNA molecules without any function) just in the beginning scene. As explained [51], due to the trade-off for a ribozyme strand to act as a catalyst and a template, the ribozyme, as an RNA species, may have found itself at a disadvantage in competition with parasites, which could act exclusively as templates. The labour division could have released the trade-off and ‘saved’ the ribozyme species. For instance, as supposed in the current context, it is circular RNAs that may have taken the role of heredity and the competition should have no longer been unfair between the ribozyme species and the parasites.

Another advantage of involving a circular genome is to resist end-degradation of RNA molecules. In modern prokaryotes, circularity of the DNA chromosome is believed to be a strategy for resisting end-degradation, which is caused mainly by exonuclease cleavage of terminal phosphodiester bonds. RNA degradation in modern cells has been studied in detail [52], in which it was shown that exonuclease activities are apparently more prevalent than endonuclease activities. This is understandable since the terminal bonds should be more exposed in solution. However, as far as we know, there is as yet no direct evidence for the assertion that RNA’s chemical degradation (in the absence of enzymes) is also more readily at the terminal bonds. Another form of RNA end-degradation may have stemmed from the spontaneous decay of nucleotide residues at chain ends. Indeed, residues within an RNA chain, which is less exposed to the solution, may have been difficult to decay. However, terminal residues should have been subjected to decay in a non-negligible way, albeit likely to a lesser extent in comparison with the decay of free nucleotides. In practice, when considering RNA’s end-degradation, here we only assumed the spontaneous decay of end-residues, but not the potentially easier breaking of the terminal phosphodiester bonds in RNA chains. This is a conservative consideration. If both forms of end-degradation were present, the benefit of the circularization in preventing end-degradation should have been more apparent.

The involvement of a circular RNA genome in the RNA world makes the rolling-circle mode of replication feasible, which might have arisen subsequently. Indeed, so far, an intractable issue concerning the RNA world scenario seems to involve the dissociation of the complementary strand from the template to enable another round of template-directed replication. In the absence of the aid of a helicase, the separation of RNA’s double strands appears to be quite difficult, especially if the RNA chain is long. The mode of rolling-circle, which is found in viroids [22,53] and viroid-like satellite RNAs [54,55], has been proposed to avoid this difficulty via continuing strand-displacement. Wherein, in particular, viroids have been suggested to be a ‘relic’ of the ancient RNA world [56]. In fact, both experimental and theoretical work has started to explore the plausibility of such a replication mode in the RNA world [57–59]. Using a model similar to the one employed here to address this issue in the future is attractive and seems promising to provide insights into the Darwinian evolution involved.

It is understandable that limited dispersal is important for the ribozymes to spread in a naked scene – to ensure that the species could benefit from its own function (e.g. REP may favour the template-directed replication of RNA; NR may produce building blocks for RNA synthesis). This is evidenced in almost each modelling study concerning this precellular phase (e.g. Ref [27–29,45,60,61]). In the present context, the replication of the circular genome could benefit from the ribozyme(s) it encodes due to the dispersal limitation (refer to the snapshots in Figures 6, 8 and Fig. S3; the associated parameter is *P*_*MN*_, see Methods for details). In fact, in a previous study of ours, we have modelled the circular genome with four genes in RNA-based protocells [62]. The purpose of that study is to explore the plausibility of the involvement of gene-linkage to avoid ‘gene loss’ during the division of the protocells [16,63]. The protocells containing the four-gene circular genome (referred to as ‘chromosome’ therein) are shown to be able to spread by virtue of the four different functional ribozymes from distinct aspects. Self-cleavage is assumed in that model by the intergenic sequence with a hammerhead ribozyme-like function to produce these ribozymes (actually inspired by the rolling circle replication which involves the hammerhead ribozyme for producing single copies of the viroid genome through self-cleaving) [22]. The naked scene modelled here can be deemed as a ‘prequel’ of that cellular scene. In this prequel, as an encouraging outcome, we saw that even without the self-cleaving function (i.e. just with ‘random breaking or synthesis’ as mentioned already), the one-gene and two-gene circular genomes can become thriving as well (thus conforming with the implication of simplicity on the origin problem). No matter how, it is expected that a membrane boundary, as a ‘stronger version’ of dispersal limitation, may have been necessary to ensure the cooperation of more ribozymes [61,64]. That is, the genomes with more genes are likely to have emerged after the ‘major transition’ in the evolution from the naked phase to the cellular phase [18–20].

## 4. Methods

### 4.1. The events occurring in the model system

Figure 9 is a schematic of the events in each time step (i.e. Monte Carlo step), as well as the associated probabilities and factors (refer to Table 1 for their descriptions). Only the molecules within the same grid room may interact with each other. A molecule may move to an adjacent room (related probability: *P*_*MN*_ for nucleotides and RNA and *P*_*MNP*_ for nucleotide precursors).

A nucleotide precursor may form a nucleotide (randomly as A, G, C, or U) in a non-enzymatic way (*P*_*NF*_) or catalysed by NR (*P*_*NFR*_). A nucleotide may also decay into their precursors (*P*_*ND*_). Nucleotides and linear RNAs may conduct random intermolecular ligation (*P*_*RL*_) to form longer chains. A linear RNA may conduct intramolecular end-to-end ligation (*P*_*EL*_) and thus transform into a circular one. An RNA molecule may attract substrates (nucleotides or oligomers) (*P*_*AT*_) via base-pairing with some error rate (*P*_*FP*_). The attraction following an adjacent substrate that is already located on the template would be easier than the *de novo* attraction (*F*_*DA*_) (i.e. the ‘primer effect’). In addition, a linear RNA may be more difficult to act as a template to attract substrates than a circular RNA (*F*_*LT*_). The substrates aligned adjacently on the template may be ligated in a non-enzymatic way (*P*_*TL*_) or catalysed by REP (*P*_*TLR*_) – that is, the template-directed synthesis. The substrates or the full complementary chain may separate from the template (*P*_*SP*_). Phosphodiester bonds within an RNA chain may break (*P*_*BB*_) – thus a circular RNA may turn into a linear one and a linear RNA may split into fragments. A nucleotide residue at the end of an RNA chain may decay into a nucleotide precursor (*P*_*NDE*_). The REP and NR may catalyse their corresponding reaction for multiple times (*T*_*REP*_ and *T*_*NR*_) in a time step.

Notably, similar to our previous modelling work concerning the scenes in the RNA world, the energy problem is here not considered explicitly. For example, nucleotides and oligonucleotides are implicitly assumed to be activated – in particular, when they form from the degradation of RNA, they are assumed to be activated again immediately to be able to be reused in the further synthesis of RNA. In fact, such *in situ* activation has been revealed to be possible by lab work [65–67]. In reality, the energy source may have involved chemical energy in the hatchery of the primordial life, such as hydrothermal vents at the sea bottom [68–70] or hydrothermal fields on land [71,72], as supposed. Since the substrates are here assumed to be always ‘activated’, the RNA species in the model system are competing for materials but not energy – as mentioned already, the total materials in the system are limited (*T*_*NPB*_). Certainly, in reality, competitions for materials and energy are both possible in Darwinian evolution.

### 4.2. The setting of parameters

The probabilities and the factors concerning the events in the system should be set according some rules. Reactions catalysed by ribozymes should be much more efficient than corresponding non-enzymatic reactions, so *P*_*TLR*_ >> *P*_*TL*_ and *P*_*NFR*_ >> *P*_*NF*_. Template-directed ligation should be apparently more efficient than intermolecular random ligation, which should be roughly in the same scale as the intramolecular end-to-end ligation of a linear RNA, so *P*_TL_ >> *P*_RL_ ≈ *P*_*EL*_. Here, nucleotide residues within the chain are assumed to be unable to decay – they should be protected therein, whereas those at the end of the chain, which is ‘semi-protected’, decay at a rate lower than that of free nucleotides, i.e. *P*_NDE_ < *P*_ND_. The template-directed synthesis without the aid of a proteinase could not have a very high fidelity, so *P*_*FP*_ could not be set a very small value. A linear RNA should have a less templating-efficiency than a circular RNA, so *F*_*LT*_ < 1; the *de novo* attraction of a substrate onto a template may be more difficult than the attraction following a primer, so *F*_*DA*_ > 1 (see below for a detailed explanation about the ways these factors work). Other considerations may include: *P*_*BB*_ may be higher than *P*_*RL*_ but lower than *P*_*NDE*_; *P*_*MN*_ < *P*_*MPN*_; *P*_*NF*_ < *P*_*ND*_, etc.

In consideration of the computational intensity, total materials in the system (*T*_*NPB*_) are assumed obviously smaller in scale than the potential corresponding situation in reality; similarly, the characteristic RNA sequence for REP or NR assumed here (8nt) is likely to be much shorter than the corresponding ribozyme in reality. However, such simplifications are believed to be not in conflict with the fundamental mechanisms that may be reflected by the modelling.

Here, a large portion of parameter values were actually set based on our experience in previous modelling studies concerning the naked phase of the RNA world [26–29]. But notably, in principle, a machine learning-like approach may be useful to automatically explore the parameter values supporting such supposed scenes in the origin of life [73]. The default values listed in Table 1 were adopted to shape the cases for demonstrating our results. Though the outcome of a simulation may be influenced by the change of some ‘key parameters’ (e.g. refer to Figures 3 and 4), it turned out to be fairly robust against ‘moderate adjustments’ of most parameters.

### 4.3. Some detailed assumptions in consideration of relevant mechanisms

A linear RNA containing the characteristic sequence of a ribozyme can act as the ribozyme only when it is shorter than 1.5 times of the characteristic sequence, considering that too many redundant residues may seriously interfere with the folding of the catalytic domain.

An RNA template may attract a substrate (nucleotide or oligomer) at any ‘empty’ site, provided that the substrate is no longer than the empty site. The attraction with a foregoing substrate (as a ‘primer’) on the template is easier than the *de novo* attraction by a factor of *F*_*DA*_ (*F*_*DA*_ >1). A linear template would have a lower templating efficiency than a circular template by a factor of *F*_*LT*_ (*F*_*LT*_ <1). That is, for the attraction following a primer on a circular template, the corresponding probability is *P*_*AT*_; for the *de novo* attraction on a circular template, the probability is actually *P*_*AT*_/*F*_*DA*_; for the attraction following a primer on a linear template, actually *P*_*AT*_ × *F*_*LT*_; for the *de novo* attraction on a linear template, actually *P*_*AT*_ × *F*_*LT*_/*F*_*DA*_.

Notably, the setting of *F*_*LT*_ < 1 is here important for understanding the labour division between circular genomes and corresponding ribozymes. Indeed, in principle, circular RNAs may also adopt tightly folding structures (like the ones in viroids) [22]. However, circular RNA genomes in the beginning should have derived from the circularization of ribozymes (for instance, in the cases shown in Figure 2, the one-gene circular genome arises from the circularization of REP). While a ribozyme signifies a functional, folding RNA molecule, a circular RNA with the same sequence would be topologically hindered in folding, leading to a more relaxed structure. Especially, the RNA species in the early RNA world should have been relatively short; without free ends, a short circular RNA may ‘find itself difficult to fold’ by forming internal base pairs (even if feasible, likely in a quite limited mode). Then, in a two-gene circular genome, the two ‘ribozyme regions’ would almost certainly interfere with each other in respect of base-pairing – thus also could not folding tightly.

The probability of the separation of the two strands of a duplex RNA is actually assumed to be *P*_SP_^*r*^, where *r* = *n* ^1/2^, and *n* is the number of base pairs in the duplex. When *n* = 1, the probability would be *P*_SP_. With the increase of *n*, the probability would decrease (because *P*_SP_, as a probability, has a value between 0 and 1). That is, the separation of the two strands would be more difficult if the base pairs are more. The introduction of the 1/2 corresponds to the consideration that self-folding of single chains may aid the dissociation of the duplex.

With the breaking of phosphodiester bonds, an RNA molecule may degrade into shorter ones (including nucleotides). When the breaking site of the chain is at a single-chain region, the breaking rate is *P*_BB_. When the breaking site is within a double-chain region, the two parallel bonds may break simultaneously, with the probability *P*_BB_^3/2^. The adoption of the index 3/2, instead of 2, corresponds to the consideration of the synergistic effect of the two breaking events.

As mentioned already, nucleotide residues at the end of an RNA chain (either at the 3'- or 5'-end) may undergo decay. With a similar consideration to RNA’s chain-breaking above, when the chain end is in a single-chain state, the decay rate is *P*_*NDE*_; when the chain end is in a double-strand state, the two paired nucleotide residues may decay simultaneously, with a probability of *P*_*NDE*_^3/2^, also taking into account the synergistic effect.

The probability of the movement of an RNA molecule is assumed to be *P*_MN_/*m*^1/2^, where *m* is the mass of the RNA, relative to a nucleotide. This assumption represents the consideration of the effect of the molecular size on the molecular movement. The square root was adopted here according to the Zimm model, concerning the diffusion coefficient of polymer molecules in solution [74].

(Note: Source codes of the simulation program in C language can be obtained from Github – see Data Availability Statement. Besides the role of evidencing the reproducibility of the present study, the source codes present more details about the implementation of the model and may help readers to understand the simulation better)

## Supplementary Material

Supporting_Information.pdf

## Data Availability

All relevant data are within the paper and its Supporting Information files. Source codes of the simulation program can be obtained from: https://github.com/mwt2001gh/Circular-genome-at-the-very-beginning/blob/main/Fig2a-Crep-2.cpp (corresponding to the case shown in Figures 2A and 5B) and https://github.com/mwt2001gh/Circular-genome-at-the-very-beginning/blob/main/Fig7b-Crepnr.cpp (corresponding to the case shown in Figures 7B and 8).
