# Supplementary material for: Circular at the very beginning: on the initial genomes in the RNA world
Source: RNA Biol. 2024 Jul 17;21(1):17–31. doi: 10.1080/15476286.2024.2380130 (PMC11259081; doi:10.1080/15476286.2024.2380130)
Supplement: Supporting_Information.pdf [file KRNB_A_2380130_SM0459.pdf]

Supporting Information for –

Circular at the very beginning: on the initial genomes in the RNA world

Yufan Luo [1]†, Minglun Liang [1]†, Chunwu Yu [2], Wentao Ma [1]*

1. Hubei Key Laboratory of Cell Homeostasis, College of Life Sciences, Wuhan University, Wuhan 430072, China

2. College of Computer Sciences, Wuhan University, Wuhan 430072, China

† These two authors contribute equally to this study.

* Correspondence to:

Wentao Ma

College of Life Sciences,

Wuhan University,

Wuhan, 430072,

China

Email: mwt@whu.edu.cn

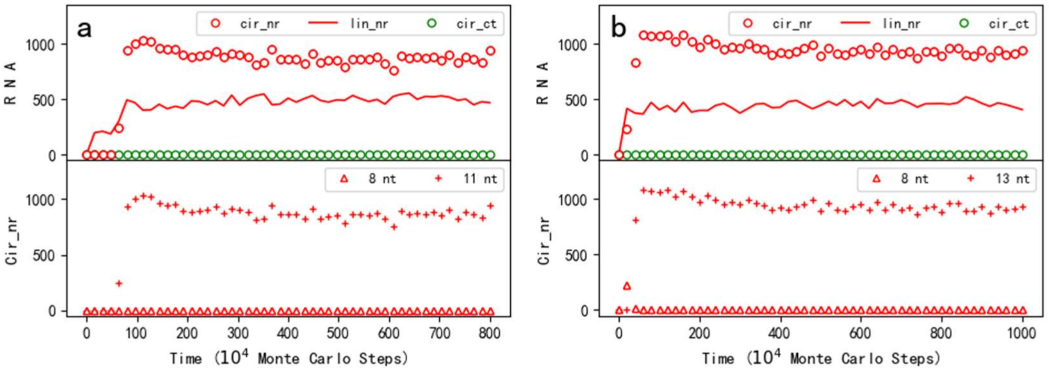

Figure S1. The spread of the circular NR genome (with a noncoding sequence). The subfigures are explained in the same way as those in Fig. 5, except that here the ribozyme involved is NR instead of REP. Legends: cir_nr – circular RNA containing the NR sequence; lin_nr – linear RNA containing the NR sequence; cir_ct – circular RNA containing the control sequence. At step 1×10^4, fifty linear RNA molecules with the NR sequence and the same number of linear RNA molecules with the control sequence are inoculated into the system. (**a**) P_{TL}=0.01, P_{NF}=0.001, and F_{LT}=0.4. (**b**) P_{TL}=0.01, P_{NF}=0.001, and F_{LT}=0.3.

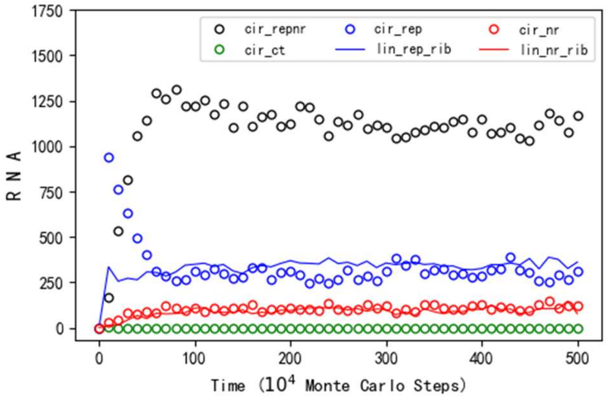

Figure S2 The deriving of a circular REP-NR genome from a circular REP-REP genome. The legends are interpreted in the same way as those in Fig. 7b. At step 1×10^4, a hundred circular RNA molecules with the REP-REP sequence (16nt in length), together with the same number of control RNA molecules (also 16nt in length, with two tandem 8nt control "ACUGACGU"), are inoculated into system. The legend "cir_ct" means circular RNA containing the 8nt control. $T_{NPB}=2\times10^5$, $P_{BB}=2\times10^{-5}$, P_{FP}=0.01, P_{MPN}=0.02, P_{NDE}=0.002, P_{NF}=0.001, and P_{TL}=0.03. See Fig. S3 for some key snapshots concerning spatial distribution of this case.

.

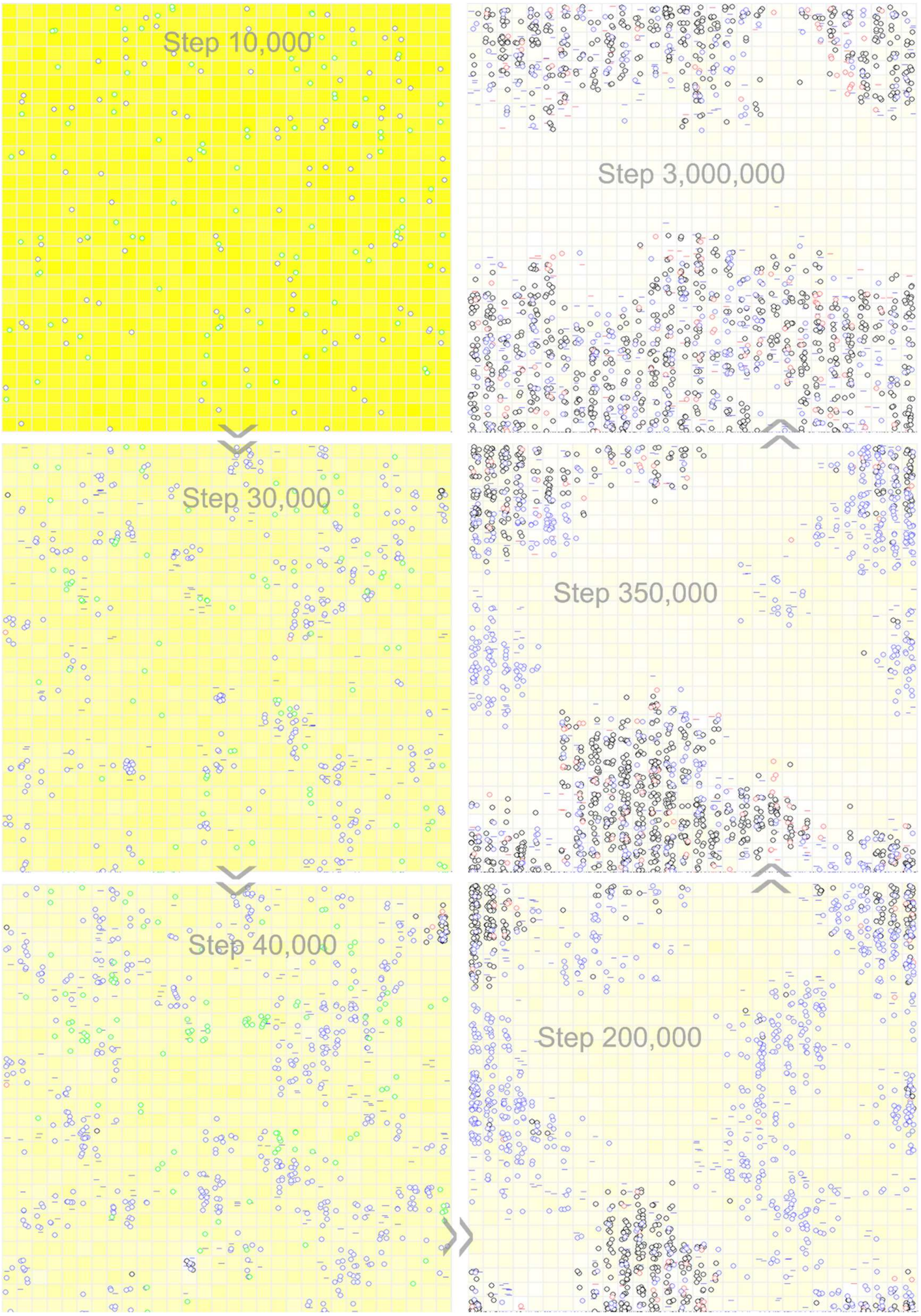

Figure S3 Snapshots showing the deriving of a circular REP-NR genome from a circular REP-REP genome. Raw materials (nucleotide precursors) are shown as yellow background, with color depth representing their quantity in the corresponding grid room. At step 10,000, a hundred circular RNA molecules with the REP-REP sequence (blue circles), together with the same number of control RNA molecules (green circles), are inoculated into system (at locations chosen randomly). The snapshot at step 30,000 shows the spread of the

REP-only genome (blue circles) and the subsequent emergence of the REP-NR circular genome (black circles, top-right and top-left). The snapshots at step 40,000, 200,000, and 350,000 shows the gradually spread of the REP-NR genome and the simultaneous decline of the REP-only genome. Red circles denote the circular NR-only genome. The snapshot at step 3,000,000 indicates the complete domination of the REP-NR genome in the system. See Fig. S2 for the evolutionary dynamics of this case.
